# The Hidden Cost of Revision Hip and Knee Arthroplasty

**DOI:** 10.1016/j.artd.2022.05.010

**Published:** 2022-06-23

**Authors:** Mackenzie A. Roof, Brett R. Levine, Ran Schwarzkopf

**Affiliations:** a NYU Langone Department of Orthopaedics, New York, NY, USA; b Rush University Medical Center, Department of Orthopaedics, Chicago, IL, USA

Revision total knee arthroplasty (rTKA) and revision total hip arthroplasty (rTHA) are technically demanding procedures. Beyond removal of primary implants and insertion of new components, revision surgeons must also manage extensive bone loss, complicated wound closures, higher infection/complication risks, and sicker patients [[1], [2], [3]]. Surprisingly, the complexity of these cases does not correlate with reimbursement rates for revision arthroplasty surgeons. Levine et al conducted 2 studies specifically investigating the relationship between case difficulty and relative value unit (RVU) compensation for rTHA and rTKA. For the hip cohort, they examined 165 primary total hip arthroplasties (pTHAs) and rTHA. When compared to the pTHA cohort, every revision type, except for head/liner exchange, reimbursed less per minute, and every revision type reimbursed less per RVU [4]. For the total knee group, they examined 154 primary total knee arthroplasties (pTKAs) and rTKA. Tibial component, all-component, and spacer revisions were reimbursed significantly less dollars per minute than pTKA. Liner exchanges and all-component revision reimbursements had fewer dollars per RVU than pTKA [5]. Overall the disparity between complexity of the cases and time dedicated to the procedures and patient care with the level of reimbursement has led to a shift in the less desirable revisions being transferred to tertiary care centers, increasing the burden on physicians at these locations. Further, the relatively favorable reimbursement for modular component exchange has led to these cases being handled locally and in some instances being performed “too frequently” based on the short operative time, low risk, and relatively high level of compensation. The disparity may lead to the unintended consequence of more washouts being done prior to a more definitive procedure for rTHA and rTKA cases.

Feng et al. modeled a dedicated rTHA service, utilizing 1 operating room, against a pTHA service with both 1 and 2 operating rooms available. Compared with a pTHA service with 1 room, revision surgeons lost 26% potential RVU per day, and compared with a 2-room pTHA service, revision surgeons lost 55% potential RVU per day [6]. The same group had similar findings in rTKA arthroplasty: with a 1-operating room pTKA, pTKA had a 1.9% RVU per day advantage over the rTKA service; however, if 2 operating rooms are used, the pTKA service generated 34.6% more RVU per day [7].

One potential explanation is that revision cases are longer. Sodhi et al. showed that the mean operative time for pTHA was 94 minutes and that for rTHA was 152 minutes. In this cohort, the mean RVU per minute was 0.260 for primary and 0.249 for revision cases, resulting in a projected $113,000 annual cost difference for a single surgeon [8].

There is also a higher infection rate in the revision cohort, contributing to operative complexity, case length, and worse patient outcomes. Samuel et al. used a septic second-stage revision as the control group and showed that the RVU per minute for the aseptic 2-component revision was 0.215 (mean operative time: 148.95 minutes); the RVU for septic, 2-component, 1-stage revision was 0.199 (mean operative time: 160.60 minutes); for septic, 2-stage revisions, the first-stage RVU per minute was 0.157 (mean operative time: 138.10 minutes); and the second-stage RVU per minute was 0.144 (mean operative time: 170.0 minutes). This analysis demonstrated that 1-component aseptic rTKA was valued the highest among these revision procedures [9].

The incidence of primary joint arthroplasty is rising, and revision cases are rising in tandem [10,11]. This trend is creating an exceedingly large demand for these procedures. We have shown that higher volume revision surgeons have better outcomes when performing rTKA than lower volume surgeons, highlighting the necessity of keeping these surgeons interested and incentivized to provide this type of service to their patients [12]. Failure to appropriately incentivize surgeons to perform these procedures may lead to a dearth of orthopaedic surgeons willing and able to take on these complicated revisions. Further work is required to reimagine the cost structure for these procedures to ensure adequate compensation for the experienced revision arthroplasty surgeon for case complexity and time invested.

One approach would be the formation of regional revision arthroplasty centers. This strategy has been successful in the trauma discipline, with patients having improved recovery at discharge and decreased mortality compared to nontrauma centers [13,14]. For patients undergoing joint replacement, these facilities would be specialized in providing care for patients undergoing revision arthroplasty procedures. Physical and occupational therapists, nurses, anesthesiologists, social workers, administrative staff members, and surgeons would encompass the care team dedicated to caring for these complicated patients, and their specialization would almost certainly streamline and enhance patient care. OrthoCarolina has already started concentrating the care of patients with periprosthetic joint infections in the southeast with their Periprosthetic Joint Infection Center. Further research is necessary to ascertain the effect of this center on both the patient and the patient- and system-level outcomes, but it is an exciting development that may pave the way for future regionalization in the world of arthroplasty.

## Conflicts of interest

Brett Levine, MD, MS is a paid consultant at Link and Exactech, receives royalties from Link, Wolters-Kluwer, SLACK Inc., and Human Kinetics, is a board member of the AAOS ALI3, and MAOA Education Committee, is the Deputy Editor of *Arthroplasty Today (AT)*, and is a member of the editorial board of the *JOA* and *AT*. He was recused from the editorial decision on this manuscript, which underwent peer review. Ran Schwarzkopf receives royalties from Smith & Nephew, is a paid consultant at Smith & Nephew and Intelijoint, holds stock at Intelijoint and PSI, receives research support from Smith & Nephew and Intelijoint, is a member of the editorial/governing board of *JOA* and *AT*, and is a board/committee member of the AAHKS and AAOS; the other author declares no potential conflicts of interest.

For full disclosure statements refer to https://doi.org/10.1016/j.artd.2019.12.004.

## Informed patient consent

The author(s) confirm that informed consent has been obtained from the involved patient(s) or if appropriate from the parent, guardian, power of attorney of the involved patient(s); and, they have given approval for this information to be published in this article.

## Appendix A Supplementary Data

Conflict of Interest Statement for Schwarzkopf

Conflict of Interest Statement for Roof

Conflict of Interest Statement for Levine
