# Supplementary material for: The Hidden Cost of Revision Hip and Knee Arthroplasty
Source: Arthroplast Today. 2022 Jun 23;16:167–8. doi: 10.1016/j.artd.2022.05.010 (PMC9249566; doi:10.1016/j.artd.2022.05.010)
Supplement: Conflict of Interest Statement for Levine [file mmc3.pdf]

<u>BLINDED</u> CONFLICT OF INTEREST STATEMENT

The Journal of Arthroplasty

(Adopted from the American Academy of Orthopaedic Surgeons disclosure statement)

The following form **<u>must be filled out completely listing all author affiliations</u>**. **<u>If no discloser is required please write/type “none” at the end of each sentence.</u>**

Manuscript Title: **The Hidden Cost of Revision Hip and Knee Arthroplasty**

---

1. Royalties from a company or supplier (The following conflicts were disclosed)
Link

2. Speakers bureau/paid presentations for a company or supplier (The following conflicts were disclosed)

3A. Paid employee for a company or supplier (The following conflicts were disclosed)

3B. Paid consultant for a company or supplier (The following conflicts were disclosed)
Link, Exactech

3C. Unpaid consultants for a company or supplier (The following conflicts were disclosed)

4. Stock or stock options in a company or supplier (The following conflicts were disclosed)

5. Research support from a company or supplier as a Principal Investigator (The following conflicts were disclosed)

6. Other financial or material support from a company or supplier (The following conflicts were disclosed)

7. Royalties, financial or material support from publishers (The following conflicts were disclosed)
SLACK Inc., Human Kinetics, Wolters-Kluwer

8. Medical/Orthopaedic publications editorial/governing board (The following conflicts were disclosed)
Deputy Editor AT; Editorial Boards—JOA and AT

9. Board member/committee appointments for a society (The following conflicts were disclosed)
MAOA Education committee; AAOS ALI3

Brett Levine, MD, MS 5/7/2022
